# Medical and Physical Intelligence

**Published:** 1826-04

**Authors:** 


					354
MEDICAL AND PHYSICAL INTELLIGENCE.
PHYSIOLOGY.
3. Experiments on the Secretion of Bile.?In order to determine
whether the bile is secreted from arterial branches or from those of the
venae portae, it is necessary to tie, either simultaneously or in succession,
the excretory ducts, and the vessels which carry both kinds of blood to
the liver. The ligature of these vessels, which has been considered im-
possible, may be easily performed upon rabbits; but, as their bile is but
lightly coloured, the results are not very conclusive. In pigeous, on the
contrary, there is some difficulty, on account of the hepatic artery; but,
as positive consequences may be drawn from them, the experiments here
related were all performed upon these birds.
1st. Ligature oj the excretory vessels. ? Ihe bile being in the course
of separation, and not being able to be evacuated, the liver swells, and
becomes filled with globules of a bright green, which colour becomes
spread over the whole surface of the liver and neighbouring parts : the
green tint is more distinct in proportion to the age of the animal, and
the length of time he outlives the operation. Tpn twenty hours
after the ligature has been applied, the animal evacuate^by the anus,
matter absolutely green, of the colour of the bile in the gorged liver,
which colour of the excrements goes on increasing in intensity until the
death of the animal; and it was found that the green matter by which
it is produced only exists in the cloaca. This fact, united to the ob-
servations of Prevost and Dumas, who have succeeded in increasing
the biliary secretion by interrupting that of the urine, demonstrates
that the kidney and liver assist each other, more or less perfectly,
respecting the excretion of their respective products, when it cannot
take place by the natural channel.
2d. Ligature of the excretory ducts and the hepatic artery.?At
the end of twelve hours, the surface of the liver receives a colour which
also tinges the neighbouring parts; the canals become filled, and an-
nounce the presence of bile. Twenty hours after the ligature, the liver
contains a great quantity of green granulations, more numerous on the
left than on the right side ; the cloaca also contains green matter, as in
the last instance. If the life of the animal is prolonged for forty hours,
the green colour oF*the liver and of the excrements becomes deeper.
These experiments appear to prove that the separation of bile follows,
and for a long time, after the liver has been deprived of arterial blood.
3d. Ligature of the hepatic artery alone.?In this case the liver does
not become gorged, because the excretory ducts are free. After death,
it is found that the secretion of bile has continued, since it is found in
the ducts; and also the matters contained in the intestines present their
usual bilious colour.
4. Ligature of the roots of the vena portas, and of the excretory
ducts.?The liver is -then directly deprived of its colour, and has only a
tint of pale rose-colour, analogous to that of the lungs of the same
Medical and Physical Intelligence. 355
birds; no trace of bile is to be met with ; the intestine contains a grey
or whitish pulp; the cloaca is full of excrement, without the least trace
or mixture of green; and, notwithstanding which, many pigeons have
lived in this state forjbirly-six hours,. Only tying the principal trunk
151 the vena portae, to permit the gastro-hepatic veins to enter, the right
lobe which receives them is, at the end of fourteen hours, in its natural
state ; whilst the left lobe is without colour, and presents on its outside
merely a few traces of bile.
From these four series of experiments, the results of which perfectly
agree with each other, it may be concluded?1st. That the ligature of
the hepatic artery does not impede the secretion of bile. 2d. That the
presence of bile becomes manifest when the excretory ducts are tied.
3d. That the blood of the vena portae is that which furnishes the ele-
ments of bile, since by tying those vessels Jhe secretion is arrested.
{Annali Universali, Deceinbre.)
SURGERY.
2. Extirpation of the Alveolar Arches of the Lower and Upper
Jaw.?Dr. Georgis Regnoli, of Forli, relates the following case,
which we have, however, greatly abridged. On returning from
Fossombrone, he was asked to see a poor woman, named Frances
Rovinella, about thirty-five years of age, and the mother of four chil-
dren, whose complaint was supposed to be incurable. The history of
her disease was as followsShe had suffered, from her infancy, violent
pains in her teeth; the first set all became carious, and, falling out in
separate pieces, were succeeded by the second dentition; these soon
became also diseased : and, added to this, she was subject to repeated
attacks of erysipelas about the head and neck. For about a year, how-
ever, the patient had escaped any attack of this kind; but, about
November 1824, she was seized with a violent pain in the head, for
which she took no remedy. At this time, whilst labouring under pain,
tumefaction, and redness of the gums, caries of the teeth and alveoli of
both jaws, she discovered that, opposite to the last molar tooth of the
right side in the lower jaw, a little tumor existed, which speedily in-
creased and ulcerated. This ulceration took possession of the alveoli
and of the gums, both within and without, leaving untouched, however,
the last molar tooth on the left sid^, which was not carious. Three
months later, a similar fungus made its appearance on the corresponding
part of the upper jaw on the right side, which spread with even greater
rapidity, not leaving any of the teeth free from it.
On examining the woman, Dr. Regnoli perceived that there was an
enormous enlargement of both the alveolar arches; a reddish and
bloody fungus, which arose from the alveoli, and from the gums inte-
riorly and exteriorly, so as to cover and hide the teeth. The alveoli
were ulcerated and soft, even down to the very roots of the teeth, both
within as well as outwardly, in both jaws; the cavity of the mouth was
much diminished by the swellings, each of them exceeding the size of
the thumb : it was easy, by means of the finger, to discover the limits
of the disease. The woman complained of pain, increased during
3&f> Medical and Physical Intelligence.
mastication; the fungous substances bled at the least touch, and poured
out a fetid ichor; and the health began to be affected. Nevertheless,
the tongue, throat, and all the soft parts, appeared to be free from
disease.
Dr. Regnoli persuaded this woman to enter into the hospital at Pesaro,
for the purpose of submitting to an operation; and after being subjected
for a few days to a proper regimen, and having been purged, the opera-
tion was performed on the 18th of May, 1825, in the presenceof several
physicians and surgeons. The patient was seated facing the light;
the head supported upon the breast of an assistant, who at the same
lime made pressure upon the labial arteries, (submaxillary ?) The
operator then divided the whole of the lower lip, and detached it in a
great measure from the lower jaw, in order to expose the fungus; he
then cut circularly the soft parts and periosteum below all the alveoli in
which the bone was healthy; and upon the fore and more prominent
part of the chin, he employed a few strokes of the saw. At this point
he inserted a strong cutting scalpel into the groove made by the
saw, which he drove in with a few blows with a hammer, first directed
to the left and then to the right side; the assistant, in the mean time,
drew the jaw downwards and forwards; and the whole osteo-sarcoma
was now removed, by merely dividing the soft parts adhering to the
iuternal and concave part of the bone. The last molar tooth on the
Jpft side was, however, left untouched, but it did not show any marks
of disease. The arterial branches, which bled profusely, were touched
with the qctual cautery, as were also the remaining parts in which any
suspicious appearance was observed. The removal of the alveolar arch
of the upper jaw was performed in the same manner; and the hemor-
rhage was arrested by the same means. The lower lip was then brought
together with three needles, united by the twisted suture. At this
tjnje the patient faiqted, but was soon restored.
The symptoms that succeeded the operation were, lor the nrst days,
severe pain in the bead, apd consequent deprivation of sleep, with fever,
$nd considerable swelling of the face; which were all overcome on
tjie sixth day, by bleeding, purging, and proper diet. On the 28th day,
the cicatrisation of the wound was complete; a few pieces of dead bone
having been removed some days previously. The appearance of the
patient presented but littje deformity, the lips turning inwards, but in
ijp great degree ; the speech is also somewhat altered; but she was able
to eat even bread and meat, was free from pain ; and the cicatrix of the
alveolar borders was hard and equal, and they could bear to be pressed
against each other without producing pain. (Ibid. Septembre.)
3. New Cupping Apparatus.?We have lately seen a simple and in-
genious contrivance for facilitating the operation of cupping in the hands
of inexperienced persons, and we have no hesitation in bearing testi-
mony to its merits; as we believe it will be found to answer all the
purpqg?s designed by the iiiventor.* It is not intended that Mr.
Clark's method should interfere with the modes already practised by
* Mr. Clark, of King-street) Holborn.
Catalogue of Medical Books. 357
expert cuppers, as, when once acquired, nothing can be more simple.
It is a notorious fact, however, that much practice is required to com-
municate sufficient dexterity in the old way ; and, unless the dexterity,
when acquired, is kept up by constant practice, it is soon forgot; so
that the country practitioner often finds himself at a loss when called
upon to perform an operation generally believed to be simple. Another
advantage possessed by Mr. Clark's invention is, that it relieves the
operation of much of its terrors, which, in children and nervous per-
sons, ought to recommend it to the consideration even of experienced
cuppers. The importance of this will appear when it is considered that
fear tends to drive the blood from the surface, and thus defeats the
object of the operation. In the army, where we understand it has
already been partially introduced, and in the navy, the new cupping
apparatus must be of use, as those unaccustomed to the operation are
certainly much less likely to fail with it than with the common cupping
class.
Mr. Clark's improvement consists in introducing into the glass a
narrow slip of silver, so thin as to be elastic, and thus readily to ac-
commodate itself to the inner surface; from the centre of this is a little
projection, which is perforated, and so contrived that a piece of silver
wire, in size and shape like a common pin, is easily fixed to it; at the
other end of the pin is a piece of sponge, or other soft substance, oil
which the spirit is to be poured. This is to be kindled, and the glass
applied while it yet burns, the vacuum resulting being more perfect the
sooner the application is effected.
i ?    -    ??i?. ? i-r
358
METEOROLOGICAL JOURNAL,
From February 20, lo March 19, 1826, inclusive.
By Messrs. William Harris and Co. Mathematical Instrument Makers,
50, Holboru, London.
Feb.
20
21
22
23
24
25
26
27
Mar-
1
2
3
4
5
6
7
8
9
10
11
12
13
14
15
16
17
18
19
Rain
gauge
29-72
30.06
29.85
29.76
30.03
29.87
30.26
30.16
30.08
29.92
29.65
29.56
29.52
29.69
30.04
29.71
29.93
30.02
30.20
30.18
30.31
30.32
29.85
29.73
30.04
30.26
30.12
29.75
29.93
30.06
29.89
29.79
30.03
30.03
30.28
29.96
30.06
29.76
29.62
29.72
29.62
30.03
29.73
29.81
29.92
30.15
30.20
30.24
30.32
30.12
29.83
29.84
30.19
30.22
29.85
29.87
UE
LUC'S
HYG.
9 A.M.
WSW
W
WSW
WSW
WNW
W
W
WSW
WSW
SWva
SSW
w
SW
SW
s
SW
s
s
E
SE
ENE
SSW
w
NE
ENE
WSW
W
10p.m.
W
SSW
SW
WNW
WSW
w
SW
SW
w
SW
SW
SW
WNW
SW
SW
SW
s
E
E
ESE
ENE
ENE
SW
NW
ENE
SSW
WSW
NW
ATMOSPHERIC
VARIATIONS.
9 A. .11.
Fine
Clond.
S.Rain
Fine
Rain
Fine
Fair
S.Kain
Fine
Rain
Fine
Fair
2 P.M.
Fine
Cloud.
Fine
Rain
Fair
Rain
Fine
Rain
Rain Fine
lOp.M.
Rain
Fine
Rain
Fine
Cloud.
Fine
Rain
Fine
Fair
Fine
The quantity of rain fallen in the month of February,
was 1 inch and 34.100ths.

				

